# P-2283. Reversing Antimicrobial Resistance (AMR) in Febrile Neutropenia. Is it Possible?

**DOI:** 10.1093/ofid/ofae631.2436

**Published:** 2025-01-29

**Authors:** Nitin Bansal, Ishita Sachdeva, Dinesh Bhurani, Narendra Agarwal

**Affiliations:** Rajiv Gandhi Cancer Institute, Delhi, Delhi, India; Washington Universoty, St. Louis, Missouri; Rajiv Gandhi Cancer Institute, Delhi, Delhi, India; Rajiv Gandhi Cancer Institute, Delhi, Delhi, India

## Abstract

**Background:**

Multi-drug resistance in gram-negative bacilli is major cause of concern in managing patients with febrile neutropenia particularly among hematology-oncology patients. Reversing AMR has never been demonstrated previously in this setting.

Table of various parameters comparing Cohort A and B

**Figure:**
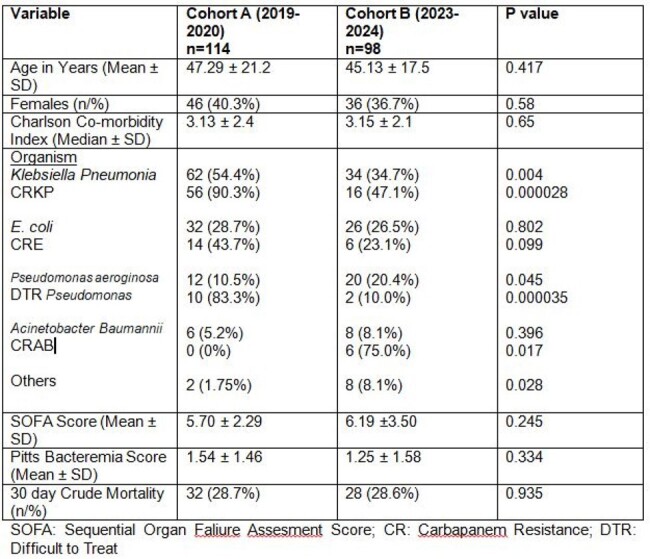

**Methods:**

This retrospective study compares clinical and microbiological outcomes of patients admitted to hematology-oncology unit of a tertiary care center in India with febrile neutropenia and gram-negative bacteremia. Patients admitted from April 2019- April 2020 were included in Cohort A and April 2023-April 2024 were included in Cohort B.

Bar diagram showing change in resistance pattern in two cohorts

**Figure:**
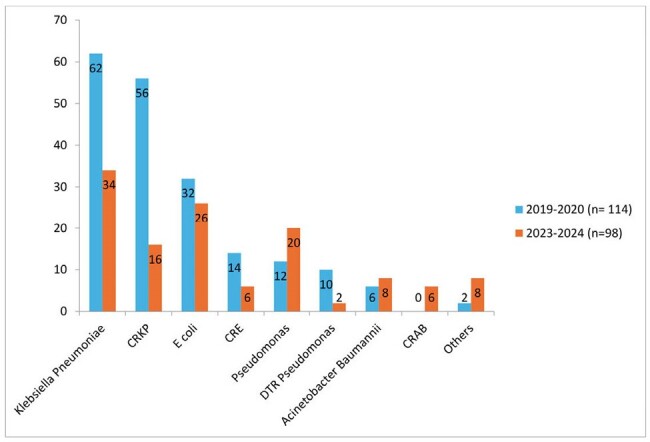

**Results:**

A total of 212 patients (Cohort A: 114, Cohort B 98) were included in this study (Table 1). Mean age (53 ± 21.2 years vs 46.5 ± 17.5; p = 0.417) and gender distribution (females: 40.3% vs 36.7%; p = 0.58) were comparable and so was distribution of malignancies. Mean Sequential Organ Failure Assessment Score (6 ± 2.29 vs 5 ±3.50; p = 0.245), mean Charlson co-morbidity Index (3 ± 2.4 vs 3 ± 2.1; p = 0.65), mean Pitts Bacteremia score (1 ± 1.46 vs 1 ± 1.58; p = 0.334) and 30 day crude mortality (28.7% vs 28.6%; p = 0.935) were not statistically significant between the two cohorts. Incidence (Figure 1)of carbapenem resistance (CR) *Klebsiella pneumoniae* (56 (90.3%) vs 16 (47.1%); p < 0.01), CR *E. coli* (14 (43.7%) vs 6 (23.1%); p = 0.099) and DTR (difficult-to-treat) *Pseudomonas aeruginosa* (10 (83.3%) vs 2 (10.0%); p < 0.01) significantly reduced in Cohort B as compared to Cohort A. Involvement of Infectious Disease (ID) service, following antibiotic stewardship (AMS) team advice, improved diagnostic facility and infection control were major interventions during the study period.

**Conclusion:**

This study highlights that incorporating ID service and following AMS activities along with other infrastructure development can reverse AMR in high risk setting like febrile neutropenia among hematology-oncology patients.

**Disclosures:**

All Authors: No reported disclosures

